# Benefit-risk balance of S-1 versus UFT as adjuvant chemotherapy for stage II/III rectal cancer (JFMC35-C1: ACTS-RC)

**DOI:** 10.1093/oncolo/oyag081

**Published:** 2026-03-15

**Authors:** Jean-Christophe Chiem, Hatem Alharazin, Everardo D Saad, Koji Oba, Masaru Muto, Hisakazu Yamagishi, Junichi Sakamoto, Takaki Yoshikawa, Marc Buyse

**Affiliations:** One2Treat, Louvain-la-Neuve, 1348, Belgium; One2Treat, Louvain-la-Neuve, 1348, Belgium; IDDI (International Drug Development Institute), Louvain-la-Neuve, 1341, Belgium; Department of Biostatistics, School of Public Health, Graduate School of Medicine, The University of Tokyo, Tokyo 113-0033, Japan; Japanese Foundation for Multidisciplinary Treatment of Cancer, Tokyo 136-0071, Japan; Japanese Foundation for Multidisciplinary Treatment of Cancer, Tokyo 136-0071, Japan; Japanese Foundation for Multidisciplinary Treatment of Cancer, Tokyo 136-0071, Japan; Gastrointestinal Medical Oncology Division, National Cancer Center Hospital, Tokyo 104‑0045, Japan; One2Treat, Louvain-la-Neuve, 1348, Belgium; IDDI (International Drug Development Institute), Louvain-la-Neuve, 1341, Belgium

**Keywords:** rectal neoplasms, S-1, UFT, generalized pairwise comparisons, prioritized outcomes, net treatment benefit, benefit-risk analysis, patient-centricity

## Abstract

**Background:**

Given the superior relapse-free survival (RFS) and different safety profiles of 1 year of adjuvant S-1 or uracil/tegafur (UFT) for stage II/III rectal cancer, the benefit-risk of these 2 regimens was formally assessed using the net treatment benefit (NTB).

**Patients and methods:**

Individual patient data from the Japanese Foundation for Multidisciplinary Treatment of Cancer (JFMC) 35-C1 trial were used. S-1 and UFT were compared regarding RFS, incidence of grade ≥3 symptoms, and incidence of grade ≥3 laboratory abnormalities reported as adverse events (AEs). Laboratory abnormalities and symptoms were analyzed as binary variables and as counts. Univariate and multivariate NTBs were computed for various ways of prioritizing the outcomes.

**Results:**

The univariate NTB for RFS was 9.2% (95% CI, 3.4%-15.2%, *P* = .005) in favor of S-1. The univariate NTB was not statistically significant for any symptom. For grade ≥3 laboratory AEs, only thrombocytopenia was statistically significant in favor of UFT (NTB = −0.8%; 95% CI, −1.6% to −0.02%; *P* = .044). In the multivariate analysis considering RFS as the outcome of first priority, the incidence of grade ≥3 symptoms as second, and the incidence of grade ≥3 laboratory abnormalities as third, the multivariate NTB was 8.8% (95% CI, 2.7%-14.9%, *P *= .014) in favor of S-1. In sensitivity analyses according to age group, the NTB was generally positive for patients <70 years but nonsignificant for those ≥70 years old.

**Conclusion:**

The reanalysis of the JFMC 35-C1 trial suggests that S-1 has a superior benefit-risk to UFT when RFS is considered as the outcome of first priority, followed by the incidence of grade ≥3 symptoms and of grade ≥3 laboratory abnormalities.

Highlights

- S-1 improves RFS vs UFT in patients with rectal cancer, but this alone does not reflect the benefit-risk balance.
- A novel statistical methodology can provide a patient-centric benefit-risk assessment called the net treatment benefit (NTB).
- The NTB prioritizing RFS > Symptoms > Lab abnormalities, shows a significant superiority of S-1.
- The NTB shows age-based difference for S-1: superiority in patients <70 but no significant difference in those ≥70.

Implications for practice

In Japan, both S-1 and UFT are chemotherapy options in the adjuvant treatment of rectal cancer. The benefit-risk balance between these 2 competing treatments can be assessed formally using generalized pairwise comparisons. When this method is used, the superiority of one of the interventions depends on how individual stakeholders prioritize efficacy and safety outcomes based on their personal preferences. Our results suggest that choosing between these 2 agents will depend on the selection and relative prioritization of outcomes that matters to patients with rectal cancer who are candidates to adjuvant chemotherapy.

## Introduction

Perioperative (ie, neoadjuvant and/or adjuvant) therapy for stage II and III rectal cancer has evolved considerably in recent years. More than a decade after the establishment of neoadjuvant chemoradiation as the standard of care for these patients,^1^ total neoadjuvant therapy—which includes neoadjuvant chemotherapy and either short-course or long-course radiation^2–4^—has gained increased acceptance in Western countries.^5^^,^^6^ Based on tumor location, risk stratification, and mismatch repair proficiency, there are currently several strategies that may include observation, short- or long-course radiation, and preoperative and/or postoperative chemotherapy.^5^ Both in Western and Eastern countries, total mesorectal excision is an integral component of treatment for stage II and III rectal cancer, based on its ability to improve local control.^7^ In Japan, the addition of lateral lymph-node dissection with autonomic nerve preservation is advocated with the aim of improving outcomes, particularly in stage III rectal cancer.^8^^,^^9^ In these cases, radiotherapy is often omitted,^9^ and adjuvant, fluoropyrimidine-based chemotherapy assumes a more prominent role with the aim of preventing distant recurrence and improving overall survival (OS).^10^

The Japanese Foundation for Multidisciplinary Treatment of Cancer (JFMC) 35-C1 trial demonstrated the superiority of 1 year of adjuvant S-1 over 1 year of uracil/tegafur (UFT) in terms of relapse-free survival (RFS) among patients with stage II and III rectal cancer not receiving preoperative therapy.^11^ The 5-year RFS was 66.4% for S-1 and 61.7% for UFT, with a hazard ratio of 0.77 (95% CI, 0.63-0.96; *P* = .0165). With regard to safety, adverse events (AEs) of all grades were reported in 82.3% of patients in the S-1 arm and 73.9% in the UFT arm, with corresponding frequencies of AEs ≥grade 3 of 13.4% and 11.7%, respectively. There were nominal differences in the incidence of specific ≥grade 3 laboratory abnormalities and symptoms reported as AEs, with thrombocytopenia (0.9% vs 0%), bilirubin increase (1.3% vs 1.0%), anorexia (2.6% vs 1.0%), diarrhea (2.6% vs 2.3%), nausea (1.3% vs 0.4%), vomiting (0.4% vs 0.2%), skin rash (0.9% vs 0.2%), and fatigue (2.1% vs 0.6%) being more frequent with S-1, and aspartate aminotransferase (AST; 0.9% vs 1.5%) and alanine aminotransferase (ALT; 0.9% vs 2.3%) increases being more frequent with UFT.

In randomized trials, the analysis of multiple outcomes such as RFS, OS, and AEs, is conventionally made using aggregate data from patients in each treatment arm and for each outcome separately. These aggregate and separate results may inform regulatory decisions and clinical practice, but they pose limitations. Firstly, results from different outcomes cannot be formally combined in a single measure of the benefit-risk balance because these outcomes are of different types (times to event for efficacy, occurrence of specific AEs for safety). Moreover, these separate analyses ignore the correlation between outcomes. For example, these analyses do not make a distinction between a scenario in which most patients who experience AEs derive no benefit from treatment and a situation in which most patients experiencing toxicity also derive a benefit. A novel method for analyzing multiple outcomes, called generalized pairwise comparisons, estimates a single measure of treatment effect called the net treatment benefit (NTB).^12^ The method can incorporate outcomes of any type, and it takes the association between outcomes into account.^13^ Given the superiority of S-1 over UFT in terms of RFS but not OS, and differences in the safety profile of both agents, we formally compared these 2 fluoropyrimidines using the NTB in an attempt to summarize the benefit-risk balance in the JFMC 35-C1 trial.^11^

## Methods

### Data source

All analyses were conducted using individual patient data from JFMC 35-C1, an open-label phase 3 trial that compared S-1 (tegafur, gimeracil, and oteracil potassium in a molar ratio of 1:0.4:1; 80-120 mg/day on days 1-28, followed by 14 days’ rest) vs UFT (500-600 mg/day on days 1-5, followed by 2 days’ rest), both for a planned duration of 1 year, in 961 patients enrolled at 222 sites in Japan between 2006 and 2009.^11^ These patients were randomized in a 1:1 ratio within 42 days after surgery using stratification using the minimization method.^14^ Institution, tumor location (above vs below the peritoneal reflection), depth of invasion (T1/2 vs T3/4), and lymph node metastasis (N0 vs N1/2) were used as minimization factors. The primary endpoint was RFS, defined as the time between the date of surgery and recurrence or death from any cause, whichever came first. Secondary endpoints were OS and AEs, the latter assessed using Common Terminology Criteria for Adverse Events, version 3.0. Since the trial was approved by the institutional review board at each institution and all patients provided written informed consent, no further ethics assessment was sought for the use of de-identified data.

### Overview of generalized pairwise comparisons

The method of generalized pairwise comparisons is summarized in the Supplementary Materials (Appendix) and described in detail elsewhere.^12^^,^^15^ In brief, the method allows the joint analysis of multiple outcomes of different types, such as continuous, categorical, and time-to-event variables, under a single statistical test and measure of treatment effect. A unique feature of the method is its allowance for different stakeholders—such as patients, physicians, trialists, and regulators—to indicate their individual order of priority among different outcomes when 2 treatments are compared in a randomized trial. Once an order of priority among outcomes of interest is defined, the 2 treatments are compared through pairwise comparisons between each patient from the experimental group and each patient from the control group. For each pair of patients thus formed, the outcomes are sequentially compared, starting from the first outcome, which is the one considered most important. Within a given pair, if one of the patients has a better result on the first outcome, the pair is classified as favorable or unfavorable to the experimental arm (in this case, S-1); otherwise, the comparison is considered neutral. Neutral pairs (or “ties”) can arise if (1) both patients in the pair present the same value for a given outcome, (2) there is lack of information for either patient (due to censoring or missing data), or (3) when the difference between both patients does not reach pre-defined thresholds of clinical similarity (see below). When a favorable or unfavorable result is observed for a given pair and a given outcome, the analysis moves on to the next pair. Otherwise, a pair is classified as neutral for that outcome, and the analysis moves on to the next outcome within the same pair (Figure S1 of the Supplementary Materials provides, as an illustrative example, the multivariate analysis carried out for the current work). As long as a pair continues to be neutral, the pairwise comparison moves on to the next outcome; whenever one of the outcomes breaks the “tie,” a pair is classified as favorable or unfavorable. The overall percentages of favorable and unfavorable pairs are then used to compute absolute or relative measures of treatment effect. Different measures of treatment effect can be obtained from such an analysis of pairwise comparisons,^12^^,^^16^^,^^17^ but here we focus on the NTB. Of note, if both efficacy and safety outcomes have been considered, the NTB formally quantifies the benefit-risk balance; importantly, the NTB is the measure of choice for benefit-risk analyses because it quantifies absolute risks and allows the separate assessment of the contribution of each outcome.^12^^,^^18^ The NTB can be interpreted as the net probability that a patient taken randomly from the experimental arm would have a better net outcome than a patient taken randomly from the control arm. Moreover, the NTB can be interpreted in terms of the number-needed-to-treat (NNT): since the NTB is a difference in absolute risks, its reciprocal yields the NNT. Another attractive property of pairwise comparisons is the possibility of using thresholds of clinical similarity, as mentioned above.^19^ If a threshold of 0 is used, any difference in outcomes between 2 patients in a pair is of interest. Alternatively, if only differences above a certain threshold (eg, a difference in *m* months in the analysis of a time-to-event outcome) is considered, this means that differences of smaller magnitudes are not considered as clinically relevant and therefore lead to a neutral result for that outcome. For statistical inference regarding the NTB, the large-sample distribution of a U-statistic, can be used.^20^

### Statistical analysis

All possible pairs of patients, one from each arm of JFMC 35-C1, were formed to conduct different analyses involving multiple outcomes: RFS; the occurrence of symptoms of grade ≥3 reported as AEs; and the occurrence of grade ≥3 laboratory abnormalities reported as AEs. Both symptoms and laboratory abnormalities were analyzed as categorical variables (as the worst grade per patient for each AE) and as counts (ie, the frequency of occurrence of different AEs; in this case, the analysis considered events of any grade, grade ≥2 events, and grade ≥3 events). These outcomes were grouped in 5 clusters: time-to-event outcomes; grade ≥3 symptom outcomes; grade ≥3 laboratory outcomes; counts of symptom events; and counts of laboratory events. Table S1 of the Supplementary Materials displays the clusters of outcomes and descriptive results by treatment arm, as well as hazard ratios for RFS and OS from conventional Cox regression models. The NTB was computed as the proportion of all favorable pairs minus the proportion of all unfavorable pairs.^12^ Before multivariate analyses, univariate GPC analyses were performed for each outcome separately to estimate a single NTB per outcome. Even though the statistical inference based on the univariate GPC yields significance levels corresponding to conventional analyses, using the NTB allows a straightforward interpretation of the treatment effect on the same absolute scale regardless of the type of event, whether it be a time-to-event variable (RFS), a categorical variable (worst grade of symptom or laboratory abnormality) or a count variable (number of symptoms or laboratory abnormalities).^21^ Two multivariate analyses were conducted: one considered RFS, the frequency of occurrence of grade ≥3 symptoms, and the frequency of occurrence of grade ≥3 laboratory abnormalities; the other considered RFS, the frequency of occurrence of grade ≥3 symptoms, grade ≥2 symptoms, and symptoms of any grade. Different sensitivity analyses were conducted with additional outcomes and separately according to age group (<70 vs ≥70 years). The analyses were stratified according to tumor location, depth of invasion, and lymph-node status. The follow-up for RFS and OS was restricted^22^ at 6 years and correction for censoring was applied.^23^ To account for multiplicity in all multivariate analyses, a sequential testing procedure was used, starting by testing the overall NTB (ie, all outcomes considered) and then testing sequentially from bottom to top of the hierarchy of outcomes until a non-significant *P*-value was found. This testing procedure preserves the overall, 2-sided type-I error at the level of 5%. All analyses were carried out using One2Treat-Insights software (One2Treat, Louvain-la-Neuve, Belgium) and a software package openly available in the Comprehensive R Archive Network.^24^

## Results

### Univariate analyses

The univariate NTB for RFS was 9.2% (95% CI, 3.4%-15.2%, *P *= .0050) in favor of S-1. This translates into an NNT of approximately 11 patients (=1/0.092). When thresholds of clinical similarity of up to 6 months were used (data not shown), the NTB varied little, but always in favor of S-1. For OS, the NTB was small and not statistically significant (NTB = 1.5%; 95% CI, −3.9 to 6.8%; *P *= .57). Figure S2 of the Supplementary Materials shows the univariate NTB for each grade ≥3 symptom. The NTB was not statistically significant for any symptom. Figure S3 of the Supplementary Materials shows the univariate NTB for each grade ≥3 laboratory AE analyzed. As expected, the NTBs were positive (in favor of S-1) for AST and ALT, negative for thrombocytopenia and bilirubin increase (in favor of UFT), and close to 0 for anemia and leukopenia. Only the NTB for thrombocytopenia was nominally significant in favor of UFT, though the effect size was quite small (NTB = −0.8%; 95% CI, −1.6 to −0.0%; *P *= .044). Figure S4 of the Supplementary Materials shows the univariate NTB for symptoms analyzed as counts (ie, considering multiple occurrences of the same AE per patient). In this case, the NTB always significantly favored UFT; for symptoms of any grade, the NTB was −19.5% (95% CI, −26.0 to −12.8%; *P *< .001). This translates into an NNT of approximately 5 (ie, 5 patients need to be treated with UFT instead of S-1 for one patient, on average, to have less symptoms on UFT than on S-1). Figure S5 of the Supplementary Materials shows the univariate NTB for laboratory abnormalities analyzed as counts. None of these analyses favored significantly S-1 or UFT.

### Multivariate analyses

**Figure 1.**
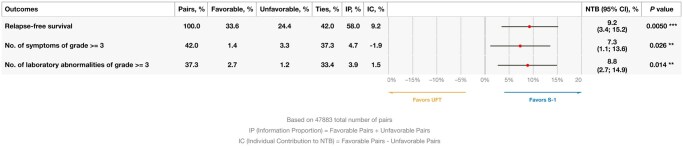
Multivariate analysis of prioritized outcomes (*P*-values are not adjusted for multiplicity; *P < .1; **P < .05; ***P < .005).

In the analysis considering RFS as the outcome of first priority, the frequency of occurrence of grade ≥3 symptoms as second, and the frequency of occurrence of grade ≥3 laboratory abnormalities as third, the NTB was 8.8% (95% CI, 2.7%-14.9%, *P *= .014) in favor of S-1 (Figure 1). This translates into an NNT of approximately 11. As shown in Figure 1, the multivariate NTB favors S-1 even though the pairwise comparisons for grade ≥3 symptoms favored UFT.

**Figure 2.**
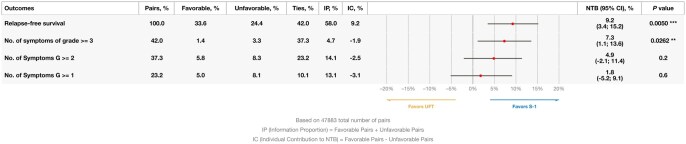
Sensitivity analysis of prioritized outcomes (*P*-values are not adjusted for multiplicity; *P < .1; **P < .05; ***P < .005).

In the analysis considering RFS as the outcome of first priority, and the frequency of occurrence of grade ≥3 symptoms, grade ≥2 symptoms, and symptoms of any grade as second, third, and fourth priorities, the NTB was no longer significant (NTB = 1.8%; 95% CI, −5.2% to 9.1%, *P *= .6) (Figure 2).

The results of the analyses according to age group are displayed in Table 1. In all cases, the NTB was positive for patients <70 years old but negative for those ≥70 years old. The only statistically significant results, in favor of S-1 in patients younger than 70, were the univariate NTB for RFS and the multivariate NTB for RFS, the frequency of occurrence of grade ≥3 symptoms, and the frequency of occurrence of grade ≥3 laboratory abnormalities.

**Table 1.** Sensitivity analyses according to age subgroups (symptoms always analyzed as counts).

Outcomes and age subgroups	Pairs	Favorable	Unfavorable	Ties	NTB (95% CI)	*P*-value
**RFS (univariate)**						
**Age <70 years**	26,102	36.5%	22.3%	41.2%	14.2% (6. 9% to 21.1%)	.0012
**Age ≥70 years**	3,137	27.1%	30.8%	42.1%	−3.8% (−16.7% to 9.1%)	.58
**RFS, grade ≥3 symptoms, and grade ≥3 laboratory abnormalities**						
**Age <70 years**						
**Recurrence-free survival**	26,102	36.5%	22.3%	41.2%	14.2% (6.9% to 21.1%)	.0012
**Grade ≥3 symptoms**	10,768	0.8%	3.0%	37.4%	12.0% (4.8% to 19.1%)	.0050
**Grade ≥3 laboratory abnormalities**	9,765	2.2%	1.1%	34.1%	13.1% (5.7% to 20.2%)	.0037
**Age ≥70 years**						
**Recurrence-free survival**	3,137	27.1%	30.8%	42.1%	−3.8% (−16.7% to 9.1%)	.58
**Grade ≥3 symptoms**	1,321	2.9%	3.9%	35.3%	−4.7% (−19.0% to 8.7%)	.54
**Grade ≥3 laboratory abnormalities**	1,107	3.2%	1.6%	30.5%	−3.2% (−17.6% to 10.6%)	.68
**RFS, grade ≥3 symptoms, grade ≥2 symptoms, and symptoms of any grade**						
**Age <70 years**						
**Recurrence-free survival**	26,102	36.5%	22.3%	41.2%	14.2% (6.9% to 21.1%)	.0012
**Grade ≥3 symptoms**	10,768	0.8%	3.0%	37.4%	12.0% (4.8% to 19.1%)	.0050
**Grade ≥2 symptoms**	9,764	4.8%	8.6%	24.0%	8.2% (0.2% to 16.3%)	.031
**Symptoms of any grade**	6,256	5.4%	8.2%	10.4%	5.3% (−3.2% to 14.3%)	0.22
**Age ≥70 years**						
**Recurrence-free survival**	3,137	27.1%	30.8%	42.1%	−3.8% (−16.7% to 9.1%)	.58
**Grade ≥3 symptoms**	1,321	2.9%	3.9%	35.3%	−4.7% (−19.0% to 8.7%)	.54
**Grade ≥2 symptoms**	1,107	8.9%	6.6%	19.8%	−2.4% (−17.8% to 12.9%)	.75
**Symptoms of any grade**	623	4.3%	6.9%	8.6%	−5.0% (−20.4% to 9.9%)	.52

NTB, net treatment benefit; RFS, relapse-free survival.

## Discussion

The current results show that the benefit-risk balance favors 1 year of adjuvant therapy with S-1 over UFT in stage II/III rectal cancer if RFS, grade ≥3 symptoms, and grade ≥3 laboratory abnormalities are considered as the outcomes of interest, with an NTB of 8.8% significantly in favor of S-1. If these outcomes are considered the most important, the treatment of 11 patients will on average lead to one more patient being free from relapse and from acute grade ≥3 symptoms/grade ≥3 laboratory abnormalities from their adjuvant chemotherapy.

The above results can be contrasted with those from the original results of the JFMC-35 trial.^11^ In the conventional analysis, the absolute 5-year RFS benefit was 4.7% (66.4% vs 61.7%); this translated into a hazard ratio (0.77) significantly favoring S-1, a relative difference of 23%. If the NNT is used as a metric of treatment benefit, the 5-year RFS difference indicates that approximately 21 (1/0.047) patients need to be treated with S-1 (instead of UFT) in order to have one additional patient free from an RFS event at 5 years. The NTB conveys a similar information in its being an absolute rather than a relative measure and hence more patient-relevant in its more immediate interpretation.^21^ On the other hand, the magnitude of difference was not the same in the current analysis, where RFS contributed with a 9.2% difference to the NTB. This stems from the fact that the NNT computed using conventional Kaplan–Meier analyses is based on a single time point, whereas the one shown here conveys the totality of the available data. In terms of the frequency of grade ≥3 AEs, the original analysis showed a 1.7% difference against S-1, combining symptoms and laboratory abnormalities (13.4% vs 11.7%). In the main analysis of the current work (see Figure 1), grade ≥3 symptoms favored UFT, whereas grade ≥3 laboratory abnormalities favored S-1. In this case, contrasting absolute differences (between frequencies in the conventional and the NTB analyses) is not straightforward, because the probabilities computed for the contribution of each outcome to the NTB are conditional on the non-occurrence of outcomes of higher priorities in the hierarchy (as described above and in the Appendix). For example, the increase of 1.9% of grade ≥3 symptoms from the use of S-1 applies only to pairs of patients whose comparison on RFS has resulted in a tie.

The main limitation of this study is the fact that its results are mostly applicable to settings in which adjuvant chemotherapy plays an important role in the treatment of stage II/III rectal cancer. As noted earlier, total neoadjuvant therapy plays an increasing role in the management of locally advanced rectal cancer.^5^^,^^6^^,^^25^ Notably, the current results depend on the specific order of priorities among outcomes, and other prioritizations of these same outcomes may lead to different NTBs, and therefore different conclusions about the relative merits of S-1 and UFT. Thus, the inclusion of additional outcomes leads to different views of the benefit-risk balance. For example, adding grade ≥2 symptoms and symptoms of any grade as third and fourth priorities led to an NTB that was no longer significant (see Figure 2). On the other hand, this is an inherent feature of the method, and one that can also be seen as one of its attractive features.

Also, the observed results may in part be related to the pharmacogenetic characteristics of Asian populations in terms of fluoropyrimidine metabolism, which are thought to differ from those in Caucasian populations.^26^ Therefore, had the study been conducted in Western countries, the NTB for the same outcomes and orders of priorities might well lead to different results. For example, lower absolute frequencies of each outcome in both arms could lead to different NTBs for each outcome, thus impacting the overall NTB.

The current analysis provides a nuanced and patient-centric view of the benefit-risk balance between 2 competing adjuvant treatments for locally advanced rectal cancer. It is worth emphasizing that this type of analysis differs from a conventional benefit-risk analysis because it combines results from different outcomes in a single measure of treatment effect. Based on the aggregate and separate (in statistical terms, marginal) results reported previously, S-1 was considered superior to UFT on the basis of RFS, but the safety profile favored either drug depending on the laboratory abnormalities and symptoms considered.^11^ This type of result, conventionally presented in the oncology literature, does not take into account the association between different outcomes. Thus, conventional analyses do not answer the important question of whether patients having benefit also have toxicity from treatment. Evidently, the answer to this question may have an impact on the choice of treatment.^13^ In this study, the multivariate NTB of 8.85% indicates the superiority of S-1 if RFS is considered as the outcome of first priority and the incidence of grade ≥3 symptoms and grade ≥3 laboratory abnormalities as second and third priorities, respectively. This choice is sensible because it takes into account the key efficacy indicator (RFS) as well as what may matter most to patients in terms of AEs. On the other hand, the current analysis also suggests that this superiority is restricted to patients <70 years. It should be noted that an interaction was found for RFS in the original analysis of this trial, with results favoring S-1 for patients <70 years and UFT for those ≥70 years old.^11^ This interaction may underlie the results described in Table 1, with the NTB in favor of S-1 for older patients and non-significantly favoring UFT for older patients. All these results should be interpreted with the caution typically warranted in retrospective subgroup analyses. The method of generalized pairwise comparisons lends itself to be used prospectively. Clinical trials aimed at estimating the NTB are under way, for instance, to evaluate reduced intensity therapeutic strategies in elderly patients with rectal cancer (NCT06052332) and in patients with acute promyelocytic leukemia using this method.^27^^,^^28^ Studies using the NTB in the metastatic setting have also been reported.^12^^,^^29–32^

Another dimension in the interpretation of the current results is the importance of shared decision making in a complex clinical setting such as locally advanced rectal cancer. In this clinical setting, several issues can affect the decision-making process related to the type of surgery and perioperative therapy, and tools to foster shared decision making between patient and physician can be of great value.^33–35^ Moreover, there is evidence that the perception on the desirability of outcomes varies between patients and healthcare providers in rectal cancer.^36^ Of note, shared decision making was not explored here in an explicit manner, given the retrospective nature of the current analysis, but considerable work has been done and is under way to explore the value of the GPC method for elicitation of patient preferences and shared decision-making.^15^

In summary, the current results show that the benefit-risk relationship favors 1 year of adjuvant therapy with S-1 over UFT in stage II/III rectal cancer if RFS, grade ≥3 symptoms, and grade ≥3 laboratory abnormalities are considered as the outcomes of interest. If these outcomes are considered as the most important, the treatment of 11 patients will on average lead to one more patient being free from relapse and from acute grade ≥3 symptoms/grade ≥3 laboratory abnormalities from their adjuvant chemotherapy.

## Supplementary Material

oyag081_Supplementary_Data

## Data Availability

The data underlying this article will be shared on reasonable request to the corresponding author.

## References

[1] Sauer R , Becker H, Hohenberger W, et al Preoperative versus postoperative chemoradiotherapy for rectal cancer. N Engl J Med. 2004;351:1731-1740. 10.1056/NEJMoa040694 15496622

[2] Bahadoer RR , Dijkstra EA, van Etten B, et al Short-course radiotherapy followed by chemotherapy before total mesorectal excision (TME) versus preoperative chemoradiotherapy, TME, and optional adjuvant chemotherapy in locally advanced rectal cancer (RAPIDO): a randomised, open-label, phase 3 trial. Lancet Oncol. 2021;22:29-42. 10.1016/S1470-2045(20)30555-6 33301740

[3] Conroy T , Bosset JF, Etienne PL, et al Neoadjuvant chemotherapy with FOLFIRINOX and preoperative chemoradiotherapy for patients with locally advanced rectal cancer (UNICANCER-PRODIGE 23): a multicentre, randomised, open-label, phase 3 trial. Lancet Oncol. 2021;22:702-715. 10.1016/S1470-2045(21)00079-6 33862000

[4] Jin J , Tang Y, Hu C, et al Multicenter, randomized, phase III trial of short-term radiotherapy plus chemotherapy versus long-term chemoradiotherapy in locally advanced rectal cancer (STELLAR). J Clin Oncol. 2022;40:1681-1692. 10.1200/JCO.21.01667 35263150 PMC9113208

[5] National Comprehensive Cancer Network. NCCN Practice Guidelines in Oncology. Rectal Cancer – v.4.2024. Accessed 4 October 2024. https://www.nccn.org/professionals/physician_gls/pdf/rectal.pdf

[6] Scott AJ , Kennedy EB, Berlin J, et al Management of locally advanced rectal cancer: ASCO guideline. J Clin Oncol. 2024;42:3355-3375. 10.1200/JCO.24.01160 39116386

[7] van Gijn W , Marijnen CA, Nagtegaal ID, et al Preoperative radiotherapy combined with total mesorectal excision for resectable rectal cancer: 12-year follow-up of the multicentre, randomised controlled TME trial. Lancet Oncol. 2011;12:575-582. 10.1016/S1470-2045(11)70097-3 21596621

[8] Sugihara K , Kobayashi H, Kato T, et al Indication and benefit of pelvic sidewall dissection for rectal cancer. Dis Colon Rectum. 2006;49:1663-1672. 10.1007/s10350-006-0714-z 17041749

[9] Tsukamoto S , Fujita S, Ota M, et al Long-term follow-up of the randomized trial of mesorectal excision with or without lateral lymph node dissection in rectal cancer (JCOG0212). Br J Surg. 2020;107:586-594. 10.1002/bjs.11513 32162301

[10] Petersen SH , Harling H, Kirkeby LT, Wille-Jorgensen P, Mocellin S. Postoperative adjuvant chemotherapy in rectal cancer operated for cure. Cochrane Database Syst Rev. 2012;2012:CD004078. 10.1002/14651858.CD004078.pub2 22419291 PMC6599875

[11] Oki E , Murata A, Yoshida K, et al A randomized phase III trial comparing S-1 versus UFT as adjuvant chemotherapy for stage II/III rectal cancer (JFMC35-C1: ACTS-RC). Ann Oncol. 2016;27:1266-1272. 10.1093/annonc/mdw162 27056996 PMC4922318

[12] Buyse M. Generalized pairwise comparisons of prioritized outcomes in the two-sample problem. Stat Med. 2010;29:3245-3257. 10.1002/sim.3923 21170918

[13] Buyse M , Saad ED, Peron J, et al The net benefit of a treatment should take the correlation between benefits and harms into account. J Clin Epidemiol. 2021;137:148-158. 10.1016/j.jclinepi.2021.03.018 33774140

[14] Coart E , Bamps P, Quinaux E, et al Minimization in randomized clinical trials. Stat Med. 2023;42:5285-5311. 10.1002/sim.9916 37867447

[15] Buyse M , Verbeeck J, De Backer M, Deltuvaite-Thomas V, Saad ED, Molenberghs G. Handbook of Generalized Pairwise Comparisons: Methods for Patient-Centric Analyses. Taylor and Francis; 2025.

[16] Pocock SJ , Ariti CA, Collier TJ, Wang D. The win ratio: a new approach to the analysis of composite endpoints in clinical trials based on clinical priorities. Eur Heart J. 2012;33:176-182. 10.1093/eurheartj/ehr352 21900289

[17] Brunner E , Vandemeulebroecke M, Mutze T. Win odds: an adaptation of the win ratio to include ties. Stat Med. 2021;40:3367-3384. 10.1002/sim.8967 33860957

[18] Verbeeck J , De Backer M, Verwerft J, et al Generalized pairwise comparisons to assess treatment effects: JACC review topic of the week. J Am Coll Cardiol. 2023;82:1360-1372. 10.1016/j.jacc.2023.06.047 37730293

[19] Peron J , Roy P, Ozenne B, Roche L, Buyse M. The net chance of a longer survival as a patient-oriented measure of treatment benefit in randomized clinical trials. JAMA Oncol. 2016;2:901-905. 10.1001/jamaoncol.2015.6359 27124210

[20] Bebu I , Lachin JM. Large sample inference for a win ratio analysis of a composite outcome based on prioritized components. Biostatistics. 2016;17:178-187. 10.1093/biostatistics/kxv032 26353896 PMC4679075

[21] Saad ED , Zalcberg JR, Peron J, Coart E, Burzykowski T, Buyse M. Understanding and communicating measures of treatment effect on survival: Can we do better? J Natl Cancer Inst. 2018;110:232-240. 10.1093/jnci/djx179 29933439

[22] Piffoux M , Ozenne B, De Backer M, Buyse M, Chiem J-C, Péron J. Restricted net treatment benefit in oncology. J Clin Epidemiol. 2024;170:111340. 10.1016/j.jclinepi.2024.111340 38570079

[23] Peron J , Buyse M, Ozenne B, Roche L, Roy P. An extension of generalized pairwise comparisons for prioritized outcomes in the presence of censoring. Stat Methods Med Res. 2018;27:1230-1239. 10.1177/0962280216658320 27487842

[24] Ozenne B , Peron J. BuyseTest: implementation of the generalized pairwise comparisons. R Package Version 2.3.10. 2022;

[25] Audisio A , Gallio C, Velenik V, et al Total neoadjuvant therapy for locally advanced rectal cancer. JAMA Oncol. 2025;11:1045-1054. 10.1001/jamaoncol.2025.2026 40638097 PMC12246951

[26] Chuah B , Goh BC, Lee SC, et al Comparison of the pharmacokinetics and pharmacodynamics of S-1 between Caucasian and East Asian patients. Cancer Sci. 2011;102:478-483. 10.1111/j.1349-7006.2010.01793.x 21143703

[27] Saúde-Conde R , Vandamme T, De Backer M, et al Efficacy and safety of short-course radiotherapy versus total neoadjuvant therapy in older rectal cancer patients: a randomised pragmatic trial (SHAPERS). ESMO Gastrointest Oncol. 2024;4:100067. 10.1016/j.esmogo.2024.100067 41648032 PMC12836652

[28] Backer M , Sengar M, Mathews V, et al Design of a clinical trial using generalized pairwise comparisons to test a less intensive treatment regimen. Clin Trials. 2024;21:180-188. 10.1177/17407745231206465 37877379 PMC11195000

[29] Peron J , Roy P, Ding K, Parulekar WR, Roche L, Buyse M. Assessing the benefit-risk of new treatments using generalised pairwise comparisons: the case of erlotinib in pancreatic cancer. Br J Cancer. 2015;112:971-976. 10.1038/bjc.2015.55 25688740 PMC4366896

[30] Peron J , Roy P, Conroy T, et al An assessment of the benefit-risk balance of FOLFIRINOX in metastatic pancreatic adenocarcinoma. Oncotarget. 2016;7:82953-82960. 10.18632/oncotarget.12761 27765912 PMC5347744

[31] Peron J , Giai J, Maucort-Boulch D, Buyse M. The benefit-risk balance of nab-paclitaxel in metastatic pancreatic adenocarcinoma. Pancreas. 2019;48:275-280. 10.1097/MPA.0000000000001234 30629024

[32] Chamseddine AN , Oba K, Buyse M, et al Impact of follow-up on generalized pairwise comparisons for estimating the irinotecan benefit in advanced/metastatic gastric cancer. Contemp Clin Trials. 2021;105:106400. 10.1016/j.cct.2021.106400 33866004

[33] Ivatury SJ , Suwanabol PA, Roo AC. Shared decision-making, sphincter preservation, and rectal cancer treatment: identifying and executing what matters most to patients. Clin Colon Rectal Surg. 2024;37:256-265. 10.1055/s-0043-1770720 38882940 PMC11178388

[34] Pieterse AH , Kunneman M, van den Hout WB, et al Patient explicit consideration of tradeoffs in decision making about rectal cancer treatment: benefits for decision process and quality of life. Acta Oncol. 2019;58:1069-1076. 10.1080/0284186X.2019.1594363 30971150

[35] Wu RC , Boushey RP, Scheer AS, et al Evaluation of the rectal cancer patient decision aid: a before and after study. Dis Colon Rectum. 2016;59:165-172. 10.1097/DCR.0000000000000528 26855389

[36] Smits LJH , van Lieshout AS, Debets S, et al Patients’ perspectives and the perceptions of healthcare providers in the treatment of early rectal cancer; a qualitative study. BMC Cancer. 2023;23:1266. 10.1186/s12885-023-11734-0 38129790 PMC10740344

