# Supplementary material for: Benefit-risk balance of S-1 versus UFT as adjuvant chemotherapy for stage II/III rectal cancer (JFMC35-C1: ACTS-RC)
Source: Oncologist. 2026 Mar 15;31(4):oyag081. doi: 10.1093/oncolo/oyag081 (PMC13033234; doi:10.1093/oncolo/oyag081)
Supplement: oyag081_Supplementary_Data [file oyag081_supplementary_data.zip › Supplement_Data/Chiem et al - Supplementary Appendix-Fig 10NOV25_clean.pdf]

Figure S1. Multivariate GPC analysis of three prioritized outcomes (relapse-free survival, number of symptoms grade ≥3 and number of laboratory abnormalities). Shown for each outcome are the definition of favorable results, the percentage of favorable, neutral and unfavorable pairs, the contribution of each outcome to the Net Treatment Benefit (NTB), and the cumulative NTB. The row "overall" displays combined results from the three outcomes and the overall NTB. Percentages are calculated with respect to the total number of pairwise comparisons.

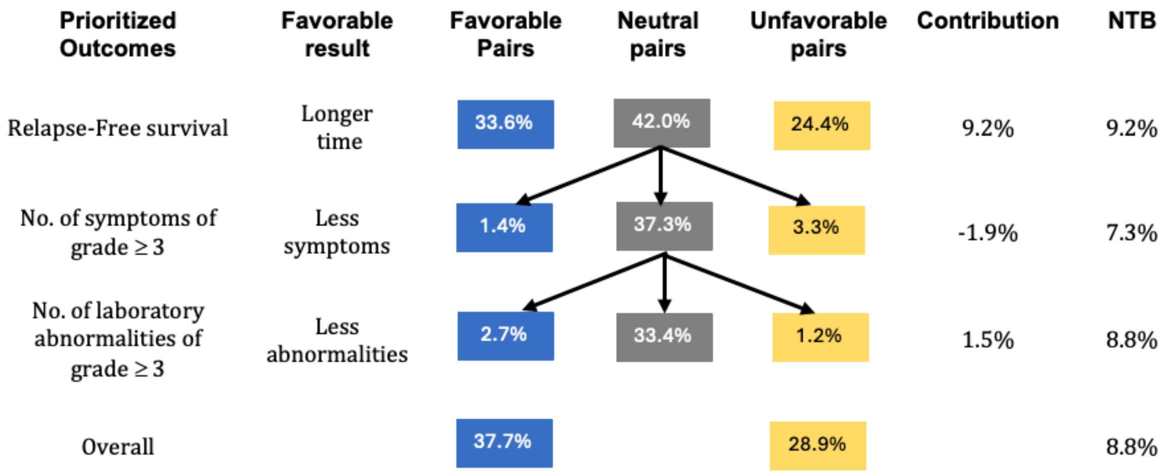

Figure S2. Univariate GPC for grade ≥3 symptoms reported as AEs and analyzed as binary variables.

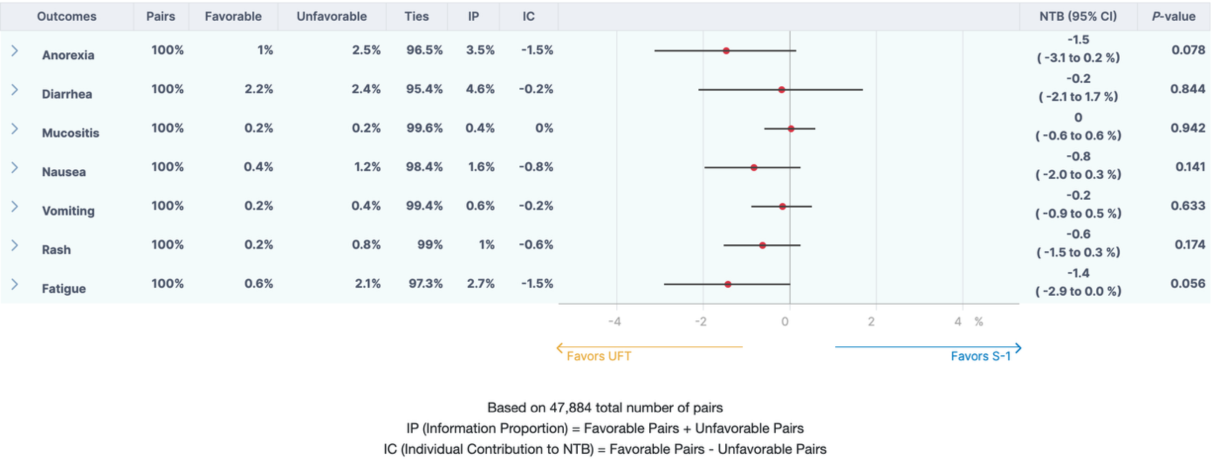

Figure S3. Univariate GPC for grade ≥3 laboratory abnormalities reported as AEs and analyzed as binary variables.

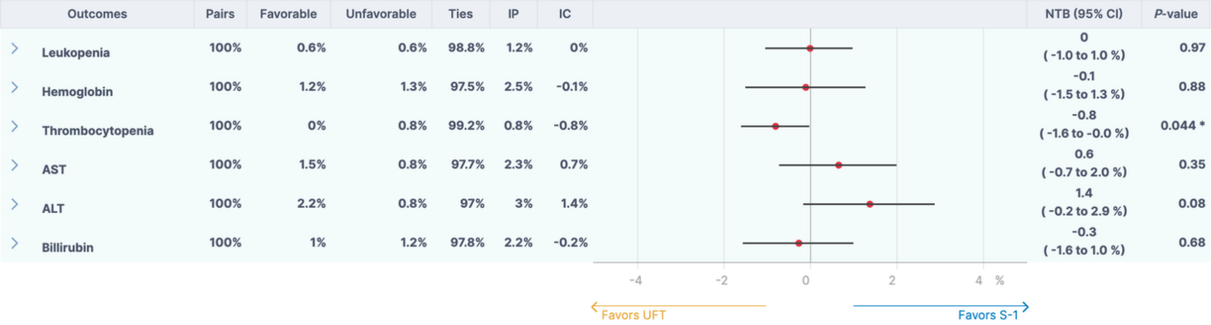

Outcomes	Pairs	Favorable	Unfavorable	Ties	IP	IC	NTB (95% CI)	P-value
Leukopenia	100%	0.6%	0.6%	98.8%	1.2%	0%	0 (-1.0 to 1.0 %)	0.97
Hemoglobin	100%	1.2%	1.3%	97.5%	2.5%	-0.1%	-0.1 (-1.5 to 1.3 %)	0.88
Thrombocytopenia	100%	0%	0.8%	99.2%	0.8%	-0.8%	-0.8 (-1.6 to -0.0 %)	0.044 *
AST	100%	1.5%	0.8%	97.7%	2.3%	0.7%	0.6 (-0.7 to 2.0 %)	0.35
ALT	100%	2.2%	0.8%	97%	3%	1.4%	1.4 (-0.2 to 2.9 %)	0.08
Billirubin	100%	1%	1.2%	97.8%	2.2%	-0.2%	-0.3 (-1.6 to 1.0 %)	0.68

Based on 47,884 total number of pairs
IP (Information Proportion) = Favorable Pairs + Unfavorable Pairs
IC (Individual Contribution to NTB) = Favorable Pairs - Unfavorable Pairs

Figure S4. Univariate GPC for symptoms analyzed as counts (i.e., considering multiple occurrences of the same AE per patient).

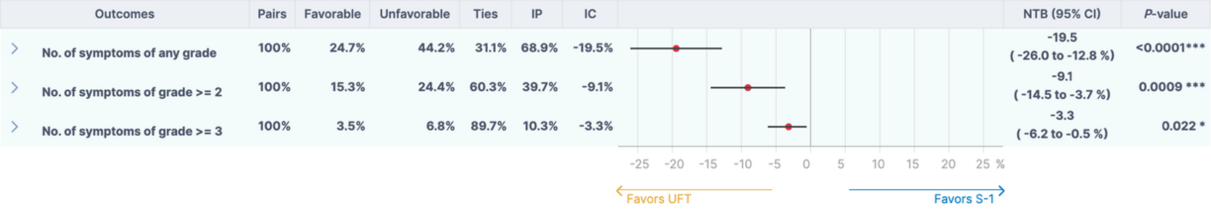

Outcomes	Pairs	Favorable	Unfavorable	Ties	IP	IC	NTB (95% CI)	P-value
No. of symptoms of any grade	100%	24.7%	44.2%	31.1%	68.9%	-19.5%	-19.5 (-26.0 to -12.8 %)	<0.0001***
No. of symptoms of grade >= 2	100%	15.3%	24.4%	60.3%	39.7%	-9.1%	-9.1 (-14.5 to -3.7 %)	0.0009 ***
No. of symptoms of grade >= 3	100%	3.5%	6.8%	89.7%	10.3%	-3.3%	-3.3 (-6.2 to -0.5 %)	0.022 *

Based on 47,884 total number of pairs
IP (Information Proportion) = Favorable Pairs + Unfavorable Pairs
IC (Individual Contribution to NTB) = Favorable Pairs - Unfavorable Pairs

Figure S5. Univariate GPC for laboratory abnormalities analyzed as counts (i.e., considering multiple occurrences of the same AE per patient).

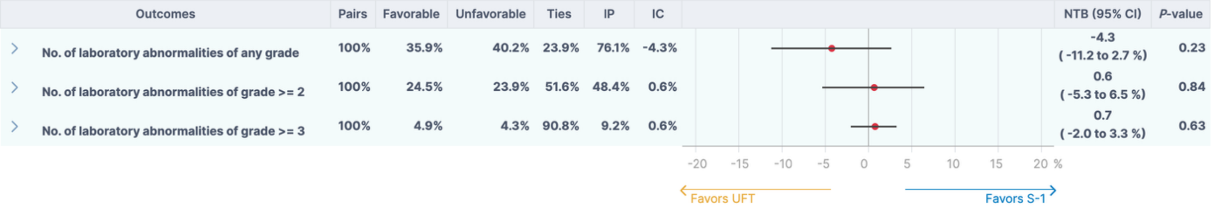

Outcomes	Pairs	Favorable	Unfavorable	Ties	IP	IC	NTB (95% CI)	P-value
No. of laboratory abnormalities of any grade	100%	35.9%	40.2%	23.9%	76.1%	-4.3%	-4.3 (-11.2 to 2.7 %)	0.23
No. of laboratory abnormalities of grade >= 2	100%	24.5%	23.9%	51.6%	48.4%	0.6%	0.6 (-5.3 to 6.5 %)	0.84
No. of laboratory abnormalities of grade >= 3	100%	4.9%	4.3%	90.8%	9.2%	0.6%	0.7 (-2.0 to 3.3 %)	0.63

Based on 47,884 total number of pairs
IP (Information Proportion) = Favorable Pairs + Unfavorable Pairs
IC (Individual Contribution to NTB) = Favorable Pairs - Unfavorable Pairs
