# Supplementary material for: Benefit-risk balance of S-1 versus UFT as adjuvant chemotherapy for stage II/III rectal cancer (JFMC35-C1: ACTS-RC)
Source: Oncologist. 2026 Mar 15;31(4):oyag081. doi: 10.1093/oncolo/oyag081 (PMC13033234; doi:10.1093/oncolo/oyag081)
Supplement: oyag081_Supplementary_Data [file oyag081_supplementary_data.zip › Supplement_Data/Chiem et al - Supplementary Appendix - Text 10NOV25_clean.pdf]

Appendix 1

Generalized Pairwise Comparisons (GPC) and the Net Treatment Benefit (NTB)

Pairwise comparisons

We wish to compare a group of patients receiving some new intervention (called "experimental") with a group of patients receiving standard of care (called "control"). Instead of comparing group-level statistics such as means, proportions or hazard rates, we use generalized pairwise comparisons (GPC), which consists in comparing each patient of the experimental group with each patient of the control group by forming all possible pairs of patients, taking one from each group (Buyse 2010). This approach is a straightforward extension of the Mann-Whitney form of the Wilcoxon test (Buyse and Molenberghs 2025). We start by considering the simple case of one outcome (or endpoint). Denote this outcome by X for patients in the experimental group and by Y for patients in the control group. A pairwise score (u_{ij}) is defined as follows for the pairwise comparison of the ith patient in the experimental group with the jth patient in the control group:

$$u_{ij} = \begin{cases} +1 \text{ if } X_i \succ Y_j \\ -1 \text{ if } X_i \prec Y_j \\ \;\;0 \text{ otherwise} \end{cases}$$

where the symbols " $\succ$ " and " $\prec$ " denote "better" and "worse" outcomes, respectively. For continuous variables, better and worse outcomes are defined simply as larger or smaller values, as the case may be; for instance, for the concentration of circulating tumor DNA, a smaller value is preferable to a larger value. For binary variables, one value of the variable defines the better outcome; for instance response to treatment or absence of toxicity (Buyse et al. 2021). For ordinal variables, smaller values are preferable to larger ones; for instance, grades of toxicity measured on the common toxicity criteria 0-5 scale. For time-to-event variables, better and worse outcomes are defined as larger or smaller values, as the case may be; for instance, for overall survival time, a larger value is preferable to a smaller one. Thus, the pairwise score u_{ij} is computed for each pair. It is set to 1 if the pair favors experimental ("favorable pair" or "win"), to -1 if the pair favors control ("unfavorable pair" or "loss"), and to 0 if the pair favors neither experimental nor control ("neutral pair" or "tie") (Buyse 2010; Pocock *et al.* 2012).

In designs that are stratified for important characteristics (e.g., age, gender, stage of disease, etc.), the comparisons are performed within each stratum. Thus, the pairwise score (u_{kij}) for the ith patient in the experimental group and the jth patient in the control group within the kth stratum is defined as follows:

$$u_{kij} = \begin{cases} +1 \text{ if } X_{ki} \succ Y_{kj} \\ -1 \text{ if } X_{ki} \prec Y_{kj} \\ \quad 0 \text{ otherwise} \end{cases}$$

Thresholds of clinical similarity

For ordinal and continuous outcomes, as well as for times to event, a threshold of clinical similarity can be specified, with any difference smaller than this threshold ignored in pairwise comparisons (Buyse 2010). For example, a difference of less than 3 months in overall survival might be considered too small to be of clinical relevance. In this case, the definition of the pairwise score is defined as follows for the ith patient in the experimental group and the jth patient in the control group:

$$u_{ij} = \begin{cases} +1 \text{ if } X_i - Y_j > \tau \\ -1 \text{ if } X_i - Y_j < -\tau \\ \ 0 \text{ otherwise} \end{cases}$$

where " τ " denotes the threshold of clinical similarity.

Multiple prioritized outcomes

Generalized pairwise comparisons are particularly useful to compare two treatment groups in terms of several prioritized outcomes. The method is stepwise: every pair is classified on the outcome of highest priority; if the pair is neutral, in other words not classified on the outcome of highest priority, then it is classified on the next prioritized outcome, and this process is repeated until the pair is classified or the pair remains neutral for the outcome of lowest priority. Mathematically, assume we have two outcomes, and assign subscript "1" to the outcome of higher priority and subscript "2" to the outcome of lower priority. Now denote $\boldsymbol{X}$ and $\boldsymbol{Y}$ the vectors of outcomes in the experimental group and in the control group, respectively. The pairwise score is defined as follows for the ith patient in the experimental group and the jth patient in the control group:

$$u_{ij} = \begin{cases} +1 \text{ if } X_{1,i} \succ Y_{1,j} \text{ or } (X_{1,i} \asymp Y_{1,j} \text{ and } X_{2,i} \succ Y_{2,j}) \\ -1 \text{ if } X_{1,i} \prec Y_{1,j} \text{ or } (X_{1,i} \asymp Y_{1,j} \text{ and } X_{2,i} \prec Y_{2,j}) \\ \ 0 \text{ otherwise} \end{cases}$$

where the symbol " $\asymp$ " denotes clinical similarity. This pairwise score can be extended to any number of prioritized outcomes of any type. The method was first proposed by Moyé et al. (1992) to combine mortality with another endpoint. Finkelstein and Schoenfeld (1999) suggested its use in AIDS to combine mortality with outcomes such as longitudinal measurements of CD4 cell counts. Pocock et al. (2012) proposed GPC (under the name "win ratio") as an alternative to a composite endpoint capturing the time to first event in cardiovascular diseases. The method has received a lot of attention because it focuses on the time to worst event rather than on the time to first event, which may be more clinically

relevant. Of note, GPC automatically takes the correlations between the outcomes into account, whereas marginal analyses of the outcomes ignore these correlations (Buyse *et al.* 2021).

Multiple non-prioritized outcomes

In some situations, it may be difficult to prioritize the outcomes of interest. In such cases, O'Brien (1984) suggested to use GPC on the sum of all outcomes (possibly weighted). A pairwise score is now defined as above for each of the outcomes; specifically, the pairwise score for the kth outcome can be defined, for the ith patient in the experimental group and the jth patient in the control group, by:

$$v_{ij}(k) = \begin{cases} +1 \text{ if the pair is favorable for the } k^{th} \text{ outcome} \\ -1 \text{ if the pair is unfavorable for the } k^{th} \text{ outcome} \\ 0 \text{ otherwise.} \end{cases}$$

The (weighted) overall pairwise score is defined as

$$u_{ij} = \frac{\sum_{k=1}^{K} w(k)\, v_{ij}(k)}{\sum_{k=1}^{K} w(k)}$$

where the weight *w(k)* reflects the relative clinical importance of the kth outcome. To avoid subjective choices, all weights are often taken equal, *i.e.*, $w(k) = \frac{1}{K}$ for $k = 1,..,K$. Note that this GPC analysis, in contrast to the GPC analysis of prioritized outcomes, does not account for the correlation between the outcomes.

Measures of treatment effect

When the GPC method is used, a natural effect measure is the Net Treatment Benefit (NTB). The estimated NTB is the mean pairwise score, i.e., the sum of all pairwise scores divided by the number of pairs:

$$NTB = \sum_{i=1}^{n_E}\sum_{j=1}^{n_C} u_{ij} \Big/ n_E . n_C$$

where n_E denotes the number of patients in the experimental group and n_C the number of patients in the control group. Denote F the number of favorable pairs ("wins"), U the number of unfavorable pairs ("losses"), and N the total number of pairs,

$$NTB = \frac{F - U}{N}$$

Pocock et al. (2012) proposed another measure of treatment effect, the win ratio (WR), defined as

$$WR = \frac{F}{U}$$

Interpretation of NTB

One advantage of NTB, is its interpretation in terms of a difference between two probabilities:

- for a single outcome, the NTB represents the net probability that a random patient has a better outcome in the experimental group than in the control group (Buyse 2010). The net probability is the difference between the probability of a better outcome in the experimental group than in the control group minus the probability of a better outcome in the control group than in the experimental group.
- for a single outcome with a threshold of clinical similarity, the NTB represents the net probability that a random patient has an outcome better by at least the threshold of clinical similarity in the experimental group than in the control group (Péron *et al.* 2016).
- for multiple prioritized outcomes, the NTB represents the net probability that a random patient has a better outcome in the experimental group than in the control group, either for the outcome of highest priority, or, in case of a tie for the outcome of highest priority, for the outcome of next priority, and so on.
- for multiple non-prioritized outcomes, the NTB represents the net probability that a random patient has a better outcome, on average, in the experimental group than in the control group.

NTB ranges from -1 to +1, with a value of 0 indicating no difference between the treatment groups. NTB being a difference between two probabilities, its reciprocal is the number needed to treat (NNT), a measure of effect familiar in health technology assessment:

$$NNT = \frac{1}{NTB}$$

Advantages of NTB as a measure of treatment effect

Tests of the null hypothesis do not depend on the measure of treatment effect; hence the so-called Finkelstein-Schoenfeld test (1999) is essentially a Wilcoxon-Mann-Whitney test. When it comes to the measure of treatment effect, NTB has clear advantages over the win ratio. From a theoretical viewpoint, NTB has desirable theoretical properties that have been studied in detail (Verbeeck et al. 2021). From an applied viewpoint, the win ratio can be misleading because it ignores ties (Butler et al. 2024); therefore, the win odds may be preferable when a large proportion of pairwise comparisons are neutral (Dong et al. 2020; Brunner et al. 2021). More importantly, NTB can be decomposed into additive contributions of prioritized outcomes, whilst the win ratio does not lend itself to such a decomposition. Mathematically, denoting F^i the number of pairs classified as favorable by the i^{th}outcome, and U^i the number of pairs classified as unfavorable by the i^{th}outcome, the overall NTB for d prioritized outcomes can be written as a sum of contributions of all outcomes:

$$NTB = \frac{F-U}{N} = \sum_{i=1}^{d} \frac{F^i - U^i}{N} = \sum_{i=1}^{d} NTB^i$$

This decomposition of NTB is crucially important from an interpretational perspective, as it quantifies the contribution of each outcome to the overall treatment effect (Buyse and Salvaggio 2025). This information mitigates the risk of an impressive NTB being due mostly to less important clinical events.

The NTB is a difference between two probabilities (an absolute measure of effect), In contrast to the win ratio which is a ratio of probabilities (a relative measure of effect). The win ratio does not have a straightforward interpretation, except for a single outcome under proportional hazards, in which case Oakes (2016) has shown that the win ratio is the reciprocal of the hazard ratio. An important advantage of the hazard ratio is that it is likely to be similar across populations having different baseline risks. This property does not hold for the win ratio when multiple outcomes are considered, not does it hold for NTB.

An example of GPC analysis

A recently published GPC analysis provides a typical example of the calculation of the NTB of a treatment to prevent severe oral stomatitis (SOM), a severe toxicity that affects about two out of three patients with locally advanced head and neck tumors treated with radiotherapy and cisplatin. A phase 3 clinical trial was conducted in 407 patients to compare an experimental treatment (the drug avasopasem manganese) with placebo (Anderson et al. 2024). Instead of comparing the incidence of SOM (grade 3 or 4 oral mucositis) between the two treatment groups, a GPC analysis was conducted with the following prioritized outcomes:

1. Grade 4 oral mucositis

2. Grade 3 oral mucositis
3. Duration of oral mucositis (in days, with a threshold of clinical similarity of 7 days)
4. Time to onset of oral mucositis (in days, with a threshold of clinical similarity of 7 days)

The GPC analysis is shown schematically in Figure 1. Each of the four outcomes contributed to NTB, with about half of the NTB (10% out of 19%) due to a reduction in grade 4 SOM, the most highly prioritized outcome considered. Note the gain in power obtained with the GPC analysis, as shown by the decreasing *P*-values when more outcomes are included in the analysis. Note also the increased clinical relevance when more outcomes are included in the analysis. Finally, the analysis of the outcome of highest priority in a GPC analysis is independent of the outcomes of lower priority; it is therefore identical to a marginal analysis. Hence, the GPC analysis adds to the analysis of the single outcome of highest priority, though using NTB as a non-parametric measure of treatment effect.

Figure 1: Illustration of GPC analysis of four prioritized outcomes

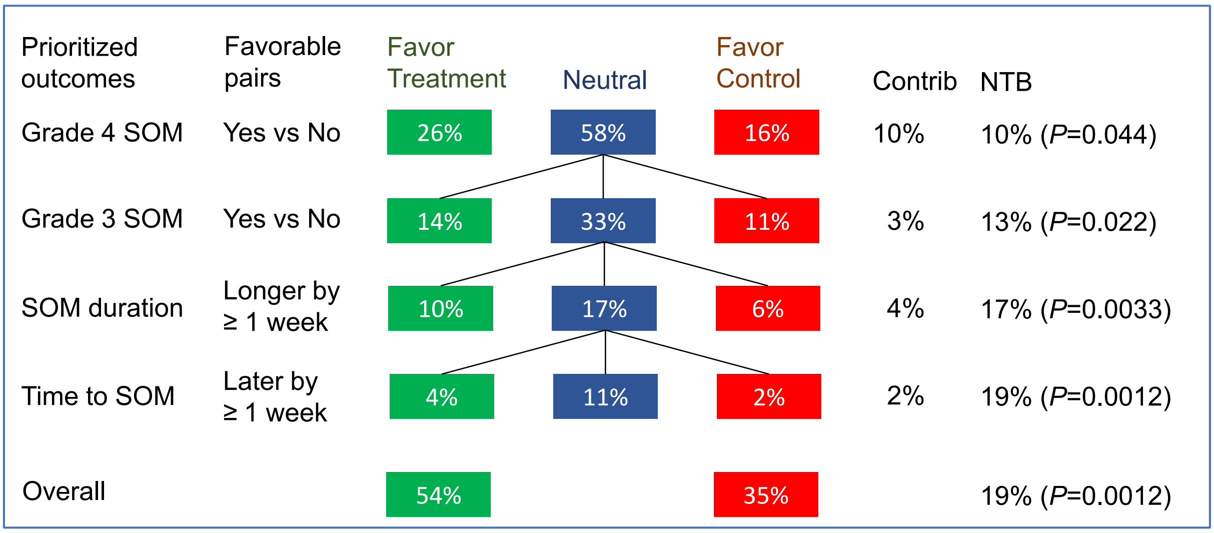

References

1. Anderson C, Salvaggio S, De Backer M, et al. (2024). Generalized pairwise comparisons to assess the Net Treatment Benefit of avasopasem manganese on reduction of severe oral mucositis in the ROMAN trial. Int J Rad Oncol, Biol, Phys, 118, e72.
2. Brunner E, Vandemeulebroecke M and Mütze T. Win odds: An adaptation of the win ratio to include ties. Stat Med 2021; 40: 3367–84.
3. Butler J, Stockbridge N, and Packer M. Win Ratio: a seductive but potentially misleading method for evaluating evidence from clinical trials. Circulation 2024; 149: 1546-48.
4. Buyse M. Generalized pairwise comparisons of prioritized outcomes in the two-sample problem. Stat Med 2010; 29: 3245-57.

5. Buyse M, Saad ED, Peron J, et al. The Net Benefit of a treatment should take the correlation between benefits and harms into account. J Clin Epidemiol 2021; 137: 148-58.
6. Buyse M and Molenberghs G. A gentle introduction to generalized pairwise comparisons. In: *Handbook of generalized pairwise comparisons. Methods for patient-centric treatment decisions* (Buyse M, Verbeeck J, De Backer M, Deltuvaite-Thomas V, Saad E and Molenberghs G, Editors). Chapman & Hall, Oxford, 2025.
7. Buyse M and Salvaggio S. Regulatory considerations for GPC analyses. In: *Handbook of generalized pairwise comparisons. Methods for patient-centric treatment decisions* (Buyse M, Verbeeck J, De Backer M, Deltuvaite-Thomas V, Saad E and Molenberghs G, Editors). Chapman & Hall, Oxford, 2025.
8. Dong G, Hoaglin DC and Qiu J. The win ratio: on interpretation and handling of ties. Biopharm Stat 2020; 12: 99–106.
9. Finkelstein D, Schoenfeld D. Combining mortality and longitudinal measures in clinical trials. Stat Med 1999; 18:1341–54.
10. Moyé LA, Davis BR and Hawkins CM. Analysis of a clinical trial involving a combined mortality and adherence dependent interval censored endpoint. Stat Med 1992; 11: 1705–17.
11. O'Brien PC. Procedures for comparing samples with multiple endpoints. Biometrics 1984; 69:1079–87.
12. Oakes D. On the win-ratio statistic in clinical trials with multiple types of event. Biometrika 2016;103: 742–5.
13. Péron J, Roy P, Ozenne B, et al. The net chance of a longer survival as a patient-oriented measure of benefit in randomized clinical trials. JAMA Oncology 2016; 2: 901-5.
14. Pocock SJ, Ariti CA, Collier TJ, Wang D. The win ratio: a new approach to the analysis of composite endpoints in clinical trials based on clinical priorities. Eur Heart J 2012; 33: 176-82.
15. Verbeeck J, Deltuvaite-Thomas V, Berckmoes B, et al. Unbiasedness and efficiency of non-parametric and UMVUE estimators of the probabilistic index and related statistics. Stat Meth Med Res 2021; 30: 747-68.
