# Supplementary material for: Benefit-risk balance of S-1 versus UFT as adjuvant chemotherapy for stage II/III rectal cancer (JFMC35-C1: ACTS-RC)
Source: Oncologist. 2026 Mar 15;31(4):oyag081. doi: 10.1093/oncolo/oyag081 (PMC13033234; doi:10.1093/oncolo/oyag081)
Supplement: oyag081_Supplementary_Data [file oyag081_supplementary_data.zip › Supplement_Data/Chiem et al - Supplementary Appendix - Table 10NOV25_clean.pdf]

Supplementary Materials to "Benefit-risk balance of S-1 versus UFT as adjuvant chemotherapy for stage II/III rectal cancer (JFMC35-C1: ACTS-RC)"

Table S1. Hazard ratios for time-to-event outcomes and descriptive results by treatment arm for the safety outcomes considered in the analysis.

Cluster	Prioritized outcomes	Treatments	
		UFT	S-1
		HR (95% CI)	
1.Survival outcomes (In Years)	Relapse-Free Survival	0.77 (0.62, 0.95)	
	Overall Survival	0.92 (0.70, 1.20)	
		N (%)	
2.Symptom outcomes of grade ≥ 3 (0 = No; 1 = Yes)	Anorexia	5 (1.0)	12 (2.6)
	Diarrhea	11 (2.3)	12 (2.6)
	Mucositis	1 (0.2)	1 (0.2)
	Nausea	2 (0.4)	6 (1.3)
	Vomiting	1 (0.2)	2 (0.4)
	Rash	1 (0.2)	4 (0.9)
	Fatigue	3 (0.6)	10 (2.1)
3.Laboratory outcomes of grade ≥ 3 (0 = No; 1 = Yes)	Leukopenia	3 (0.6)	3 (0.6)
	Hemoglobin	6 (1.3)	6 (1.3)
	Thrombocytopenia	0 (0.0)	4 (0.9)
	Aspartate aminotransferase	7 (1.5)	4 (0.9)
	Alanine aminotransferase	11 (2.3)	4 (0.9)
	Bilirubin	5 (1.0)	6 (1.3)
		Mean (SD)	
4.Count of symptoms events	Of any grade	0.89 (1.29)	1.41 (1.57)
	Of grade ≥ 2	0.31 (0.75)	0.49 (0.97)
	Of grade ≥ 3	0.05 (0.28)	0.10 (0.40)
5.Count of laboratory events	Of any grade	1.55 (1.69)	1.69 (1.76)
	Of grade ≥ 2	0.45 (0.74)	0.47 (0.78)
	Of grade ≥ 3	0.07 (0.30)	0.06 (0.29)
